# Comparative metagenomic analysis of the oral microbiome in COVID-19 patients and healthy individuals

**DOI:** 10.1038/s41598-024-81864-3

**Published:** 2025-03-25

**Authors:** Piyush Bhanu, Sakshi Buchke, Nisha Hemandhar-Kumar, Piyush Varsha, S. K. Ravi Kiran, G. Vikneswaran, Arjun Alva, G. S. Basavaraj, Jitendra Kumar

**Affiliations:** 1 Xome Life Sciences Pvt. Ltd., Bangalore Bioinnovation Centre, Helix Biotech Park, Electronic City, Bangalore, India; 2 School of Engineering, Department of Bioengineering, University of Kansas, 1450 Jayhawk Blvd, Lawrence, KS 66045 USA; 3 Morsani College of Medicine, University of South Florida, 12901 Bruce B., Downs Blvd., Tampa, FL 33612 USA; 4 Department of Neuro- and Sensory Physiology, University Medical Center Göttingen, 37073 Göttingen, Germany; 5 Saraswati Dental College, Tiwari Ganj, 233, Faizabad Rd, Uattardhona, Uttar Pradesh 226028 India; 6 Narayana Hrudayalaya Hospital, Narayana Hrudayalaya Ltd., Bommasandra Industrial Area, Hosur Road, Anekal Taluk, Bangalore, Karnataka India; 7 Biotechnology Industry Research Assistance Council (BIRAC), H728+H5G, NSIC Estate, Okhla Phase III, Okhla Industrial Estate, New Delhi, Delhi India

**Keywords:** COVID-19, SARS-CoV-2, Oral microbiome, Metagenomic sequencing, Microbial diversity, Therapeutic interventions, Long-term health, Public health strategies, Microbial dysbiosis, Pathogenicity, Probiotics, Microbiome-modulating therapies, Biotechnology, Computational biology and bioinformatics, Microbiology

## Abstract

COVID-19, caused by SARS-CoV-2, affects multiple body systems, including the oral cavity, where it may disrupt the oral microbiome in ways that contribute to disease pathology. Understanding the long-term interaction between SARS-CoV-2 and the oral microbiome is crucial, as it may reveal microbial markers valuable for diagnosing or monitoring persistent health issues in COVID-19 survivors. Metagenomic sequencing revealed significant microbial shifts in the oral microbiome of COVID-19 patients, showing reduced microbial diversity and increased prevalence of opportunistic pathogens compared to healthy individuals. Alpha diversity measures indicated lower microbial diversity and evenness, while beta diversity analyses demonstrated distinct microbial community compositions. Core microbiome analysis identified unique taxa in COVID-19 patients that may contribute to disease pathology, while differential abundance analysis highlighted specific taxa shifts, including an increase in potential pathogens. Our findings advance the understanding of microbial changes in the oral microbiome associated with COVID-19 and suggest potential targets for microbiome-based interventions. While these results indicate associations with possible health impacts, further research is needed to determine causative links and long-term implications for COVID-19 survivors. This foundational research highlights the potential for microbiome science to inform diagnostic tools, such as microbial markers for disease progression, and therapeutic approaches, including targeted probiotics, which could ultimately support better patient outcomes and public health strategies.

**Supplementary Information:**

The online version contains supplementary material available at 10.1038/s41598-024-81864-3.

## Introduction

The novel coronavirus disease 2019 (COVID-19)^1^ caused by the severe acute respiratory syndrome coronavirus 2 (SARS-CoV-2) has led to a profound global health crisis, with extensive impacts on multiple body systems, including the oral cavity^2^. SARS-CoV-2 primarily targets the respiratory system, but emerging evidence indicates interactions between SARS-CoV-2 and the oral microbiome, a complex community of microorganisms that plays a crucial role in oral and systemic health^3^. Disruption of this microbiome may contribute to various health complications, making it an important focus in understanding the disease’s pathogenesis and therapeutic potential^4^.

Compared to the respiratory and gut microbiomes, the oral microbiome has received less attention in COVID-19 research^5^. As a primary entry point for SARS-CoV-2, the oral cavity directly participates in infection dynamics, making the oral microbiome a critical yet understudied component in understanding COVID-19 pathology. The oral microbiome is also known to modulate immune responses, and disruptions caused by SARS-CoV-2 may alter immune homeostasis in the oral cavity^6^. Investigating whether COVID-19 shifts the microbial balance toward opportunistic pathogens could help reveal mechanisms underlying immune dysregulation and complications associated with the disease.

Recent studies indicate that SARS-CoV-2 may alter the composition of the oral microbiome, as suggested by its presence in saliva and its binding potential to ACE2 receptors in the oral cavity^7^. However, the specific microbial changes resulting from COVID-19 and their clinical implications are not yet fully understood. This study hypothesizes that COVID-19 is associated with distinct microbial shifts in the oral microbiome, characterized by changes in microbial diversity, composition, and the emergence of opportunistic taxa. By examining these shifts, this research aims to identify potential microbial markers for COVID-19, which could contribute to diagnostic and therapeutic strategies.

Previous research has documented various changes in the oral microbiome related to COVID-19, including alterations in microbial diversity, shifts in specific taxa, and increases in opportunistic pathogens^8,9^. For example, studies report a decrease in beneficial bacteria such as *Streptococcus* and an increase in pathogenic species like *Pseudomonas aeruginosa* in COVID-19 patients^10^. Understanding these changes is essential for identifying microbial markers that could aid in early COVID-19 diagnosis and support microbiome-based interventions.

Identifying specific microbial shifts could facilitate the development of non-invasive diagnostic tools, such as oral microbial markers for early detection and monitoring of COVID-19 progression^11,12^, as well as targeted microbiome-modulating therapies to support COVID-19 recovery. While this study aims to uncover microbial associations with COVID-19, establishing causative links requires further investigation, particularly through longitudinal and functional studies.

## Materials and methods

### Study design

**Fig. 1:**
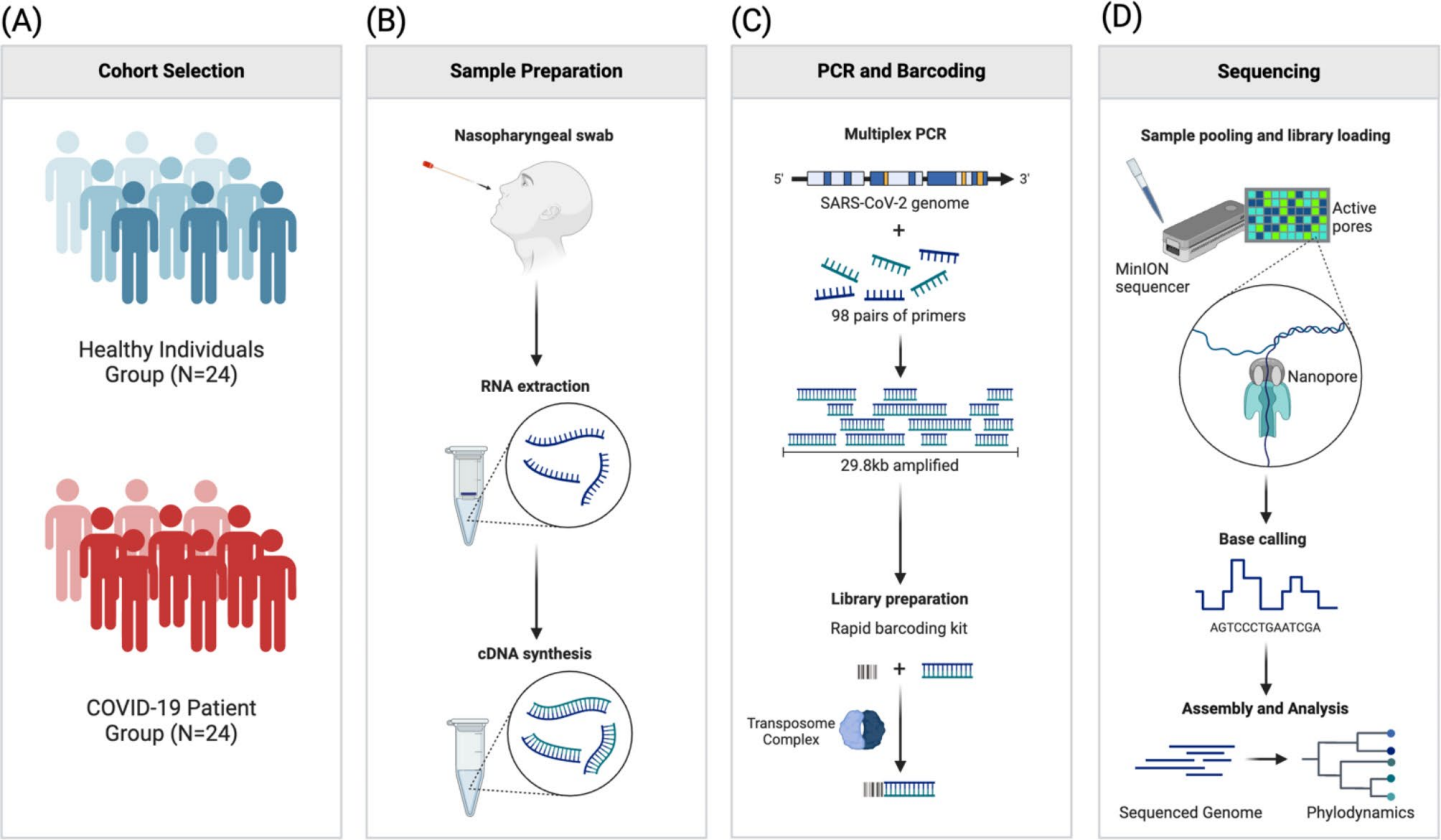
Methodology for SARS-CoV-2 Genome Sequencing Using Oxford Nanopore Technologies: This figure illustrates the detailed methodology employed in this study for SARS-CoV-2 genome sequencing, encompassing cohort selection, sample preparation, PCR amplification, barcoding, and sequencing^13^. (**A**) Cohort Selection: The study cohort was divided into two groups: 24 healthy individuals and 24 COVID-19 patients, ensuring equal representation for comparative analysis. (**B**) Sample Preparation: Nasopharyngeal swabs were collected from participants, followed by RNA extraction. The extracted RNA was then converted to complementary DNA (cDNA) via reverse transcription, preparing the samples for further amplification and sequencing. (**C**) PCR and Barcoding: The SARS-CoV-2 genome was amplified using 98 pairs of primers in a multiplex PCR, yielding a 29.8 kb amplified product. Subsequent library preparation involved the use of a rapid barcoding kit and the transposome complex to ensure efficient barcoding of the amplified products. (**D**) Sequencing: The prepared libraries were pooled and loaded onto the MinION sequencer. Sequencing was conducted using nanopore technology, followed by base calling, and the assembly and analysis of the sequenced genome, which provided detailed phylodynamic insights.

This study aims to investigate the impact of COVID-19 on the oral microbiome. Saliva was chosen as the sample type due to its ability to capture a broad representation of the oral microbiome and its ease of non-invasive collection, which is especially advantageous for large cohort studies^14,15^. Metagenomic sequencing was chosen for its ability to provide a comprehensive analysis of the entire microbial community within the oral cavity, allowing for the identification of both abundant and rare taxa and capturing shifts in microbial diversity and composition associated with COVID-19. Each stage, from sample collection to metagenomic analysis, was chosen to comprehensively capture microbial shifts associated with COVID-19, allowing us to assess the impact on microbial diversity and community composition. The study design involved several stages: sample collection, RNA extraction, sequencing, and metagenomic analysis. In this study, we divided 48 patients into two groups, namely, COVID-19 group, and non-COVID group (Fig. 1A)^16^. The sample size of 48, while insightful, limits the generalizability of the findings; larger cohorts are recommended for future studies to improve statistical power. Participants, for this study, were selected based on specific criteria, including age, gender, tobacco and alcohol use, disease state and comorbidities, and medication use, as these factors influence microbiome composition^17^. All methods were performed in accordance with the relevant guidelines and regulations. Ethical clearance for the study was obtained from the Narayana Health Academic Ethics Committee (NHAEC), registered under DGCI with the EC Registration No. ECR/772/Inst/KA/2016/RR-19, stating the permission to conduct this study at Narayana Hrudayalaya Hospital, a unit of Narayana Hrudayalaya Ltd. Written informed consent was taken from all 48 participants for this study.

### Sample collection and preparation

Oral (saliva) samples were collected from all participants using the GeneFiX™ Saliva Microbiome DNA Collector Kit (Isohelix, Cell Projects Ltd, UK). Participants were instructed to avoid eating, drinking, smoking, or brushing their teeth for at least 30 min prior to sample collection. Approximately 1 ml of saliva was collected into a tube containing stabilizing solution and shipped at room temperature for RNA extraction.

### RNA extraction, cDNA synthesis and barcoding

RNA was extracted from oral samples using the QIAamp® Viral RNA Mini Kit (QIAGEN, Cat No./ID: 52906) according to the manufacturer’s instructions. RNA quantity and quality were assessed using the Qubit RNA HS Assay (ThermoFisher, USA) and NanoDrop® spectrophotometry (Roche, USA) to ensure compatibility with sequencing requirements.

For cDNA synthesis, the SuperScript™ IV First-Strand Synthesis System (ThermoFisher, Cat No./ID: 18091050) was used following the manufacturer’s protocol. RNA was reverse transcribed into cDNA using random hexamer primers and SuperScript IV Reverse Transcriptase, as depicted in Fig. 1B. Quality control of the cDNA product included concentration measurement with Qubit, purity evaluation with NanoDrop spectrophotometry (A260/A280 and A260/A230 ratios), and optional integrity verification through gel electrophoresis.

The SARS-CoV-2 genome was amplified using 98 pairs of primers in a multiplex PCR, yielding a 29.8 kb amplified product. Unique dual barcoding and washing steps were implemented during library preparation to prevent cross-contamination, a common concern in high-throughput sequencing. Subsequent library preparation involved the use of a rapid barcoding kit and the transposome complex to ensure efficient barcoding of the amplified products (Fig. 1C).

### Sequencing

Whole genome sequencing was performed on all samples using long-read sequencing. The DNA library was prepared with the Ligation Sequencing Kit (SQK-LSK109, Oxford Nanopore Technologies, UK), loaded onto an R9.4.1 MinION flow cell (FLO-MIN106), and sequenced on the ONT MinION Mk1B device (MIN-101B). Basecalling and demultiplexing of sequence reads were performed with Guppy v4.2.2 and MinKNOW GUI v4.1.22^18^. Raw sequencing reads were stored in FastQ format for further computational analysis (Fig. 1D)^16^.

### Upstream analysis

The upstream analysis involved quality checks and quality improvement measures, including host (human) sequence removal, followed by alignment of quality-processed reads to a reference database of microbial genomes. Quality checks were performed using NanoStat (v1.4.0)^19^ and included the removal of short and sub-par quality reads (< Q5). While Phred scores of Q20 or Q30 are typically used to ensure high-quality reads, a Q5 threshold was selected in this study based on preliminary analyses showing that it provided sufficient taxonomic resolution for the oral microbiome samples. This lower threshold allowed for greater data coverage while maintaining acceptable quality for our analysis needs^20^. The 50 bp minimum read length was chosen based on pilot testing, which demonstrated that this length offered sufficient taxonomic resolution for identifying key microbial taxa in the oral microbiome dataset. This threshold was determined to balance data coverage with taxonomic accuracy for the study’s objectives.^21^. Reads shorter than 50 bp were removed, with this minimum read length based on pilot testing confirming sufficient resolution for metagenomic analysis. Suitable reads were mapped to the human reference genome (GRCh38) using Bowtie2 (v2.4.2)^22^ to filter out host sequences. Centrifuge v1.0.3^23^ was used for rapid, accurate, and sensitive microbial classification and quantification of species. A custom database built on the Reference Sequence (RefSeq) collection was used as the reference database, resulting in raw abundance profiles of prokaryotes (bacteria, archaea), eukaryotes (protozoa, metazoa, etc.), and viruses.

### Downstream metagenomic analysis

Data filtering and normalization were performed to remove low-quality or uninformative features from raw abundance data, improving downstream statistical analysis. Outliers in abundance data were identified using interquartile range (IQR)-based filtering, which is robust for handling skewed microbiome data and minimizes the influence of extreme values that may distort the overall analysis. Data filtering and normalization were performed to remove low-quality or uninformative features from raw abundance data, improving downstream statistical analysis^24^. Features with very low counts (fewer than 3 reads in less than 10% of samples) were filtered out to exclude rare taxa, focusing the analysis on microbes with consistent presence and biological relevance^25,26^. A low variance filter, using IQR variances, was applied to remove features with low variability across samples, ensuring robustness in downstream statistical analyses. Normalization addressed variability in sampling depth and data sparsity, using Trimmed Mean of M-values (TMM) for accurate biological interpretation^27^. Taxonomic composition was visualized using bar plots and pie charts to compare groups. Alpha diversity differences between the COVID-19 and non-COVID groups were assessed using the Mann–Whitney U test, a non-parametric test for comparing groups, suitable for non-normally distributed data^28^. Alpha diversity was characterized using indices like Observed, Chao1, ACE, Shannon, Simpson, and Fisher, describing the richness and evenness of the microbial communities. Beta diversity analyses employed Bray–Curtis distance and Principle Coordinate Analysis (PCoA) to visualize clustering patterns, which were evaluated using Permutational ANOVA (PERMANOVA). Alpha and beta diversity were assessed with indices (Observed, Chao1, ACE, Shannon, Simpson, Fisher) and Bray–Curtis distance, respectively, using the phyloseq package. Results were visualized as box plots or PCoA plots^29,30^.

Core Microbiome analysis identified core taxa (species) consistent across the microbial community using the R package microbiome, represented in heatmaps showing prevalence levels across detection thresholds^31^. To control the false discovery rate in the differential abundance testing across multiple taxa, the Benjamini–Hochberg correction was applied to *p*-values. This correction was selected for its ability to effectively reduce false positives, which is essential in microbiome studies involving numerous taxa comparisons^32,33^. Differential abundance analysis, performed using EdgeR v3.12 and metagenomeSeq^18^, identified significantly altered microbial abundances with adjusted *p*-values < 0.05^34–36^. EdgeR utilized RLE normalization and a Negative Binomial model for count distributions, while metagenomeSeq combined CSS normalization with zero-inflated Gaussian (FitZIG) distribution to account for under-sampling and sparsity, identifying significant features based on adjusted *p*-values < 0.05^37,38^.

## Results

This study identified distinct shifts in the oral microbiome between COVID-19 patients and healthy individuals, characterized by reduced microbial diversity and an increase in opportunistic pathogens in COVID-19 patients. We leveraged the oral microbiome sequenced data from 24 patients with COVID-19 and 24 healthy individuals from the dataset submitted to NCBI^16^. The findings are presented in several key areas: demographic and health-related characteristics, normalized abundance and diversity measures, alpha diversity, beta diversity, core microbiome composition, and differential abundance of microbial taxa. These results offer valuable insights into the microbial shifts associated with COVID-19 and highlight potential microbial markers for the disease.

### Demographic and health-related characteristics of COVID-19 and Non-COVID study groups

**Fig. 2:**
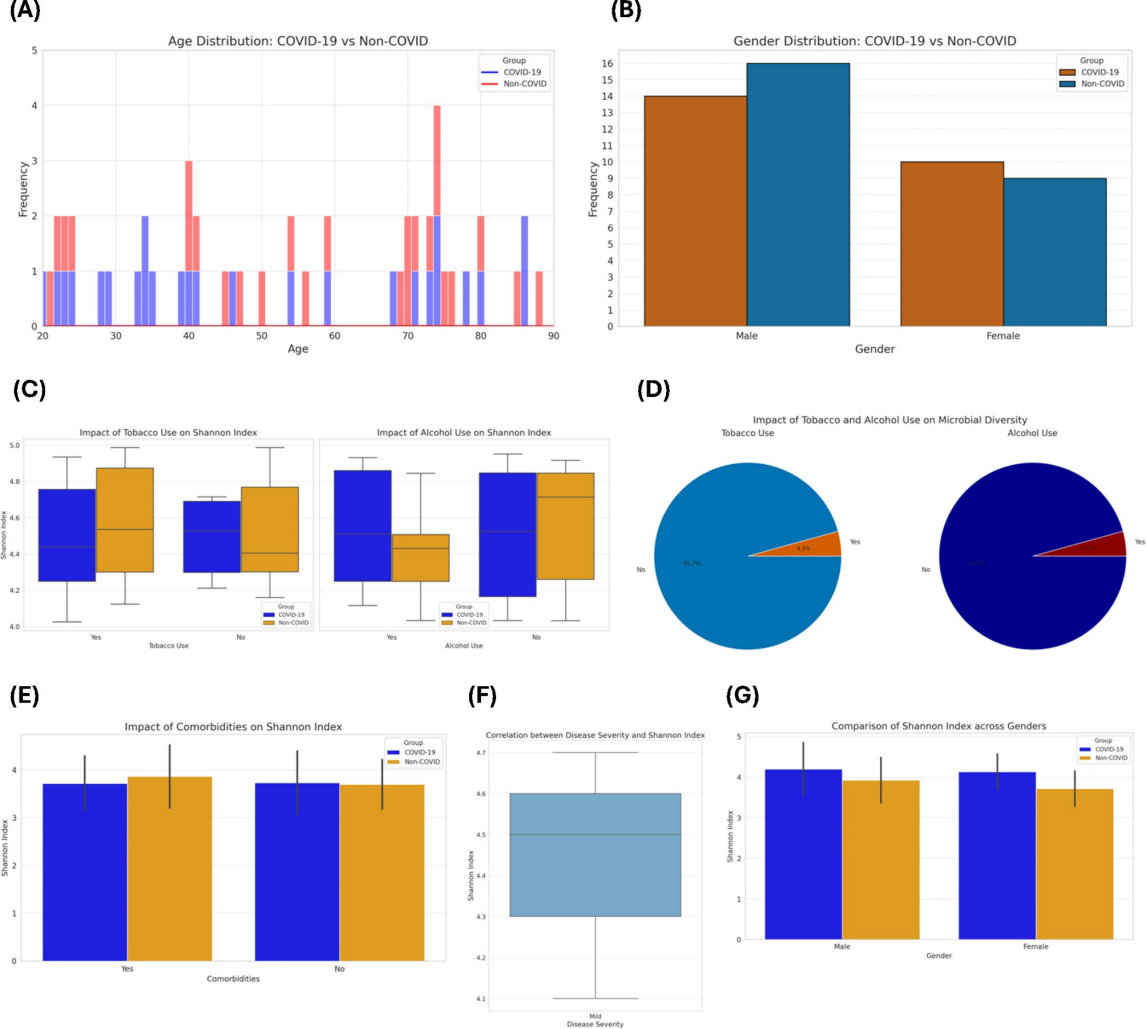
Demographic and Health-Related Characteristics of COVID-19 and Non-COVID Study Groups: Figure provides an overview of the demographic and health-related characteristics of the study groups. (**A**) Age distribution of participants across COVID-19 and non-COVID groups, illustrating the spread of ages within each group. (**B**) Gender distribution across the two groups, indicating the proportion of male and female participants. (**C**) Box plots showing the impact of tobacco and alcohol use on the Shannon diversity index in both groups, highlighting the differences in microbial diversity due to lifestyle factors. (**D**) Pie charts illustrating the prevalence of tobacco and alcohol use among participants in both groups. (**E**) Box plots showing the impact of comorbidities on Shannon diversity index, emphasizing the influence of pre-existing health conditions on microbial diversity. (**F**) Correlation between disease severity and Shannon diversity index in COVID-19 patients, depicting how disease severity affects microbial diversity. (**G**) Comparison of Shannon diversity index across genders in both groups, indicating any gender-specific differences in microbial diversity.

The age distribution chart (Fig. 2A) shows that the ages of participants in both the COVID-19 and non-COVID groups span from 20 to 90 years. The COVID-19 group exhibits a higher concentration of participants in the 30–40 age range, while the non-COVID group has a more evenly spread age distribution. This distribution is important for understanding if age-related factors could influence the microbiome differences observed in the study^39^. The gender distribution chart (Fig. 2B) indicates an almost equal representation of males and females in both groups, with slightly more males in each. This balance is crucial for ensuring that gender-specific differences do not skew the results of the microbiome analysis^40^.

Figure 2C shows the Shannon diversity index in relation to tobacco and alcohol use. COVID-19 patients who use tobacco or alcohol tend to have a lower Shannon diversity index compared to non-users, indicating that these lifestyle factors may negatively impact microbial diversity. In the non-COVID group, the impact is less pronounced but still evident^41^. The pie charts in Fig. 2D illustrate the prevalence of tobacco and alcohol use in both groups. A higher percentage of tobacco and alcohol use is observed in the COVID-19 group compared to the non-COVID group, which may contribute to the differences in microbial diversity observed^42^. Figure 2E demonstrates the impact of comorbidities on the Shannon diversity index. Participants with comorbidities in the COVID-19 group show a lower Shannon index compared to those without comorbidities. This suggests that pre-existing health conditions may exacerbate the impact of COVID-19 on microbial diversity^43^. The box plot in Fig. 2F shows a negative correlation between disease severity and Shannon diversity index among COVID-19 patients. As disease severity increases, microbial diversity tends to decrease, highlighting the significant impact of severe COVID-19 on the oral microbiome^44^. Figure 2G compares the Shannon diversity index between genders. In both groups, females exhibit a slightly higher Shannon diversity index compared to males. This gender-specific difference in microbial diversity is consistent across both COVID-19 and non-COVID groups, suggesting inherent gender-related factors influencing the microbiome^45^.

### Normalized abundance and diversity measures

**Fig. 3:**
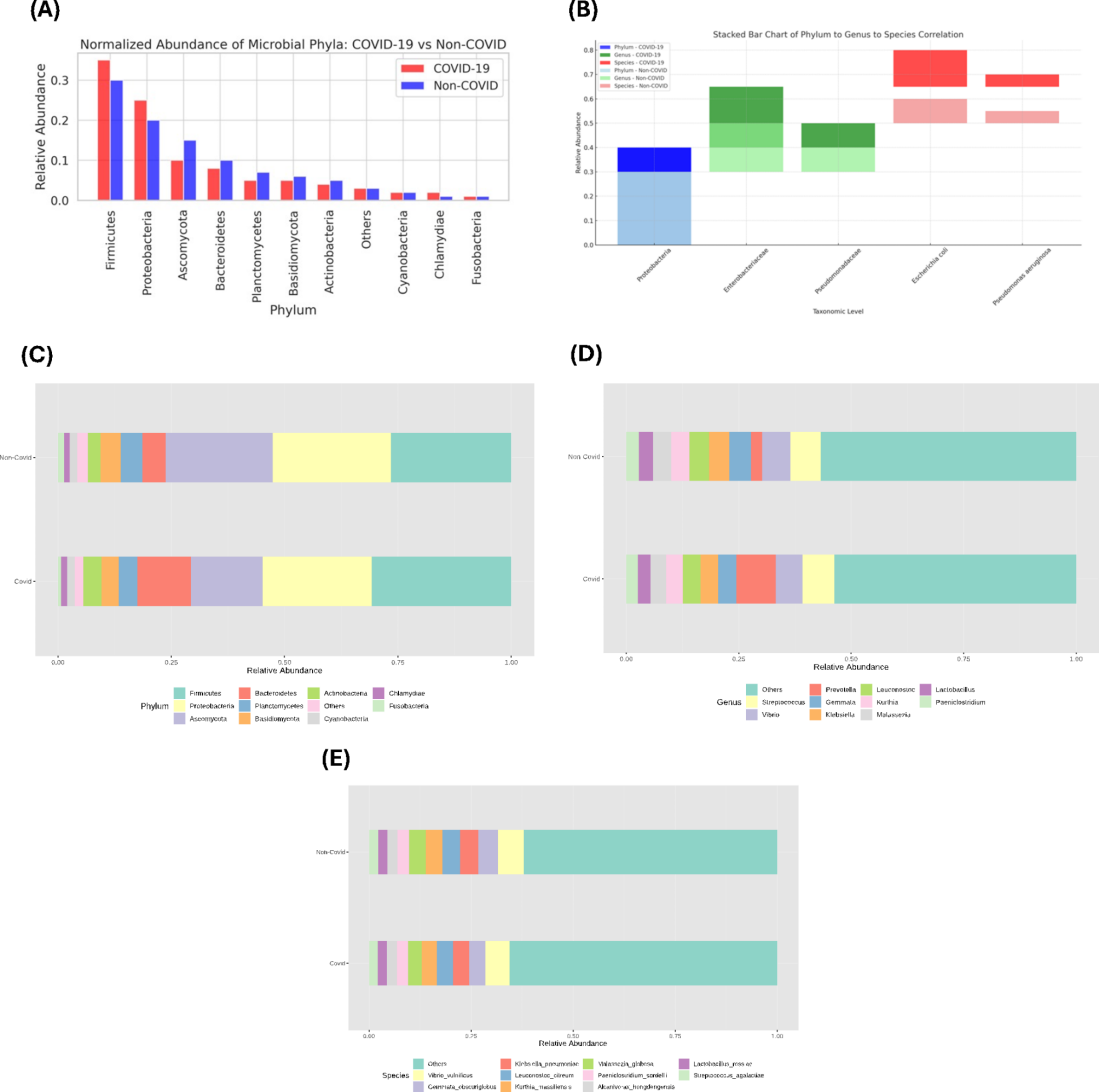
Normalized abundance and diversity measures of the oral microbiome in COVID-19 vs Non-COVID Individuals: Figure presents the normalized abundance and diversity measures of the oral microbiome in COVID-19 and non-COVID individuals. (**A**) Normalized abundance of microbial phyla, highlighting the relative abundance of different phyla in both groups. (**B**) Stacked bar chart illustrating the correlation between phylum, genus, and species levels, providing a hierarchical view of microbial composition. (**C**) Genus-level normalized abundance showing the distribution of key genera. (**D**) Phylum-level normalized abundance illustrating the distribution across different phyla. (**E**) Species-level normalized abundance depicting the distribution of specific species.

Median values were used to summarize phylum- and species-level abundances to account for the typically skewed nature of microbiome data. The median was selected as a robust measure for microbiome abundance data to minimize the influence of outliers and skewed distributions^46,47^. Outliers were identified using IQR-based filtering, and these values were retained in the analysis to reflect natural variation within the dataset. Outliers identified by IQR filtering were retained to capture the full range of variability within the microbiome community data. The Normalized Abundance of Microbial Phyla chart (Fig. 3A) shows that the relative abundance of major phyla such as *Firmicutes, Proteobacteria,* and *Bacteroidetes* differs between COVID-19 and non-COVID groups. COVID-19 patients have a higher abundance of *Firmicutes* and *Proteobacteria*, while non-COVID individuals exhibit higher levels of *Bacteroidetes*. This shift in phyla distribution suggests significant changes in the microbial landscape associated with COVID-19^48^. The Stacked Bar Chart of Phylum to Genus to Species Correlation (Fig. 3B) provides a detailed hierarchical view of the microbial composition. It highlights how changes at the phylum level propagate through to the genus and species levels. For instance, the increased abundance of *Firmicutes* in COVID-19 patients is linked to higher levels of specific genera and species within this phylum, offering a comprehensive understanding of microbial shifts. The Genus Normalized Abundance chart (Fig. 3C) shows the distribution of key genera such as *Streptococcus, Veillonella*, and *Prevotella*. COVID-19 patients exhibit higher levels of *Streptococcus* and *Veillonella*, while *Prevotella* is more abundant in non-COVID individuals. These genus-level changes align with the broader phylum-level shifts and provide more detailed insights into microbial alterations^49^. The Phylum Normalized Abundance chart (Fig. 3D) reiterates the distribution patterns observed in Fig. 3A, emphasizing the dominance of *Firmicutes* and *Proteobacteria* in COVID-19 patients and *Bacteroidetes* in non-COVID individuals. This consistency across different visualizations strengthens the validity of the observed microbial shifts. The Species Normalized Abundance chart (Fig. 3E) highlights specific species such as *Streptococcus mutans* and *Veillonella parvula* that are more abundant in COVID-19 patients. These species-level changes provide critical insights into potential microbial markers or contributors to COVID-19.

### Alpha diversity measures

**Fig. 4:**
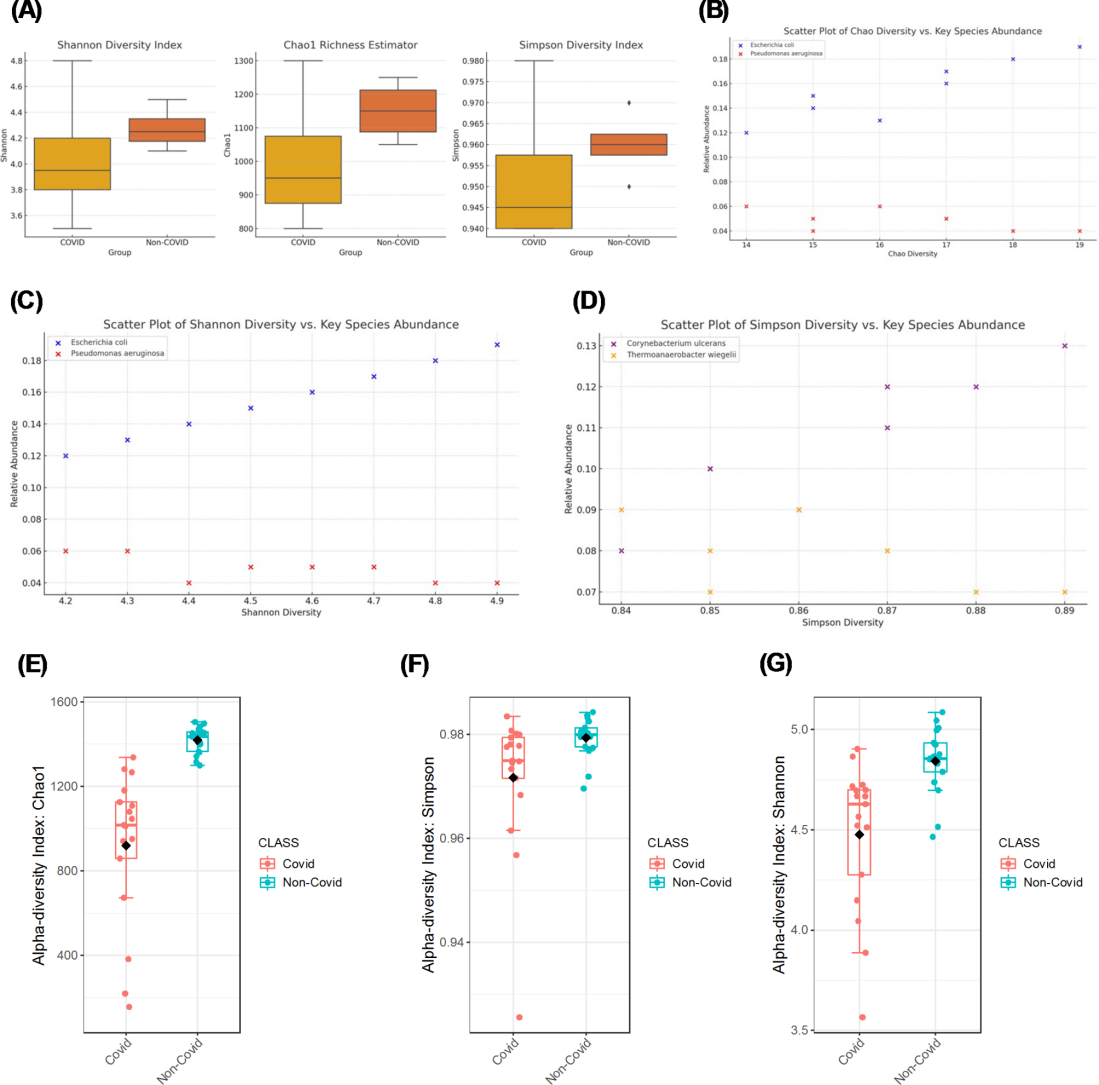
Alpha diversity measures of the oral microbiome in COVID-19 vs Non-COVID Individuals: Figure illustrates the alpha diversity measures of the oral microbiome in COVID-19 and non-COVID individuals. (**A**) Box plots of Shannon diversity index, Chao1 richness estimator, and Simpson diversity index, comparing microbial diversity within samples across both groups. (**B**) Scatter plot of Chao1 diversity index vs. key species abundance, showing the relationship between species richness and the abundance of key species. (**C**) Scatter plot of Shannon diversity index vs. key species abundance, highlighting the relationship between microbial diversity and key species abundance. (**D**) Scatter plot of Simpson diversity index vs. key species abundance, depicting the relationship between microbial evenness and the abundance of key species. (**E**) Detailed alpha diversity index for Chao1, illustrating species richness differences. (**F**) Detailed alpha diversity index for Simpson, indicating differences in microbial evenness. (**G**) Detailed alpha diversity index for Shannon, showing differences in microbial diversity.

Alpha diversity differences between the COVID-19 and non-COVID groups were assessed using the Mann–Whitney U test, appropriate for non-parametric data^28^. The Mann–Whitney U test, a non-parametric test, was chosen due to the lack of normal distribution in the alpha diversity data. The Shapiro–Wilk test was used to assess the normality of alpha diversity indices, which indicated a non-normal distribution, justifying the use of the Mann–Whitney U test for group comparisons^50^. The box plots (Fig. 4A) present the Shannon diversity index, Chao1 richness estimator, and Simpson diversity index for COVID-19 and non-COVID groups. The Shannon index measures overall microbial diversity, while the Chao1 index estimates species richness, and the Simpson index assesses evenness^51^. COVID-19 patients show a significantly lower Shannon diversity index, indicating reduced microbial diversity compared to non-COVID individuals. The Chao1 richness estimator also shows lower species richness in COVID-19 patients, suggesting a decrease in the number of unique species present. The Simpson diversity index indicates reduced evenness in the microbial community of COVID-19 patients.

The scatter plot (Fig. 4B) shows the relationship between the Chao1 diversity index and the abundance of key species such as *Escherichia coli* and *Pseudomonas aeruginosa*. Higher abundances of these species are associated with lower Chao1 diversity, suggesting that their proliferation may contribute to reduced species richness in COVID-19 patients. The scatter plot (Fig. 4C) illustrates the relationship between the Shannon diversity index and the abundance of key species. Similar to the Chao1 diversity index, higher abundances of specific species are correlated with lower Shannon diversity, indicating that the dominance of certain taxa negatively impacts overall microbial diversity. The scatter plot (Fig. 4D) depicts the relationship between the Simpson diversity index and the abundance of key species. The negative correlation observed suggests that an increase in the abundance of dominant species reduces microbial evenness, further supporting the findings from the Shannon and Chao1 indices^52^.

The detailed alpha diversity index for Chao1 (Fig. 4E) provides a comprehensive view of species richness differences between the groups. COVID-19 patients consistently exhibit lower Chao1 indices, reinforcing the observation of reduced species richness. The detailed alpha diversity index for Simpson (Fig. 4F) highlights differences in microbial evenness. COVID-19 patients show lower Simpson indices, indicating that the microbial community is less evenly distributed and more dominated by a few species. The detailed alpha diversity index for Shannon (Fig. 4G) corroborates the findings of reduced microbial diversity in COVID-19 patients. This index provides a holistic measure of diversity, incorporating both richness and evenness, and consistently shows lower values for COVID-19 patients.

### Beta diversity analysis

**Fig. 5:**
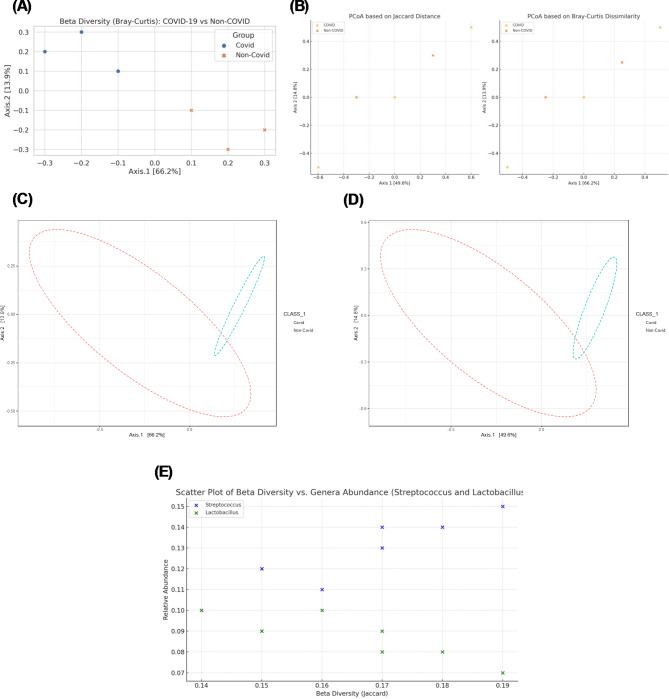
Beta Diversity Analysis of the Oral Microbiome in COVID-19 vs Non-COVID Individuals: Figure presents the beta diversity analysis of the oral microbiome in COVID-19 and non-COVID individuals. (**A**) Beta diversity (Bray–Curtis) plot, comparing the microbial community composition between the two groups. (**B**) PCoA plots based on Jaccard distance and Bray–Curtis dissimilarity, illustrating the clustering of samples and the separation between COVID-19 and non-COVID groups. (**C**) PCoA (Bray–Curtis) PERMANOVA results, providing statistical validation of the observed differences. (**D**) PCoA (Jaccard) ANOSIM results, offering additional statistical validation. (**E**) Scatter plot of beta diversity vs. genera abundance (Streptococcus and Lactobacillus), highlighting the relationship between community composition and the abundance of key genera.

The Beta Diversity (Bray–Curtis) plot (Fig. 5A) illustrates the differences in microbial community composition between COVID-19 and non-COVID groups^53^. The distinct separation between the two groups indicates significant dissimilarities in their oral microbiomes, suggesting that COVID-19 affects the overall structure of the microbial community. The PCoA plots (Fig. 5B) based on Jaccard distance and Bray–Curtis dissimilarity further highlight the separation between COVID-19 and non-COVID groups. The clear clustering of samples within each group demonstrates consistent differences in microbial community composition, with COVID-19 patients showing distinct microbial profiles compared to non-COVID individuals.

The PCoA (Bray–Curtis) PERMANOVA results (Fig. 5C) provide statistical validation for the observed differences in microbial community composition. The significant PERMANOVA results confirm that the differences between COVID-19 and non-COVID groups are not due to random variation but are statistically robust, indicating a true effect of COVID-19 on the oral microbiome. The PCoA (Jaccard) ANOSIM results (Fig. 5D) offer additional statistical validation. The significant ANOSIM results further support the conclusion that the observed differences in microbial community composition between COVID-19 and non-COVID groups are statistically significant and meaningful^54^.

The Scatter Plot of Beta Diversity vs. Genera Abundance (*Streptococcus* and *Lactobacillus*) (Fig. 5E) shows the relationship between beta diversity measures and the abundance of key genera. The plot reveals that higher abundances of *Streptococcus* and *Lactobacillus* are associated with greater dissimilarity in microbial community composition. This suggests that the presence of these genera may be driving the observed differences in beta diversity between COVID-19 and non-COVID individuals^55^.

### Core microbiome composition

**Fig. 6:**
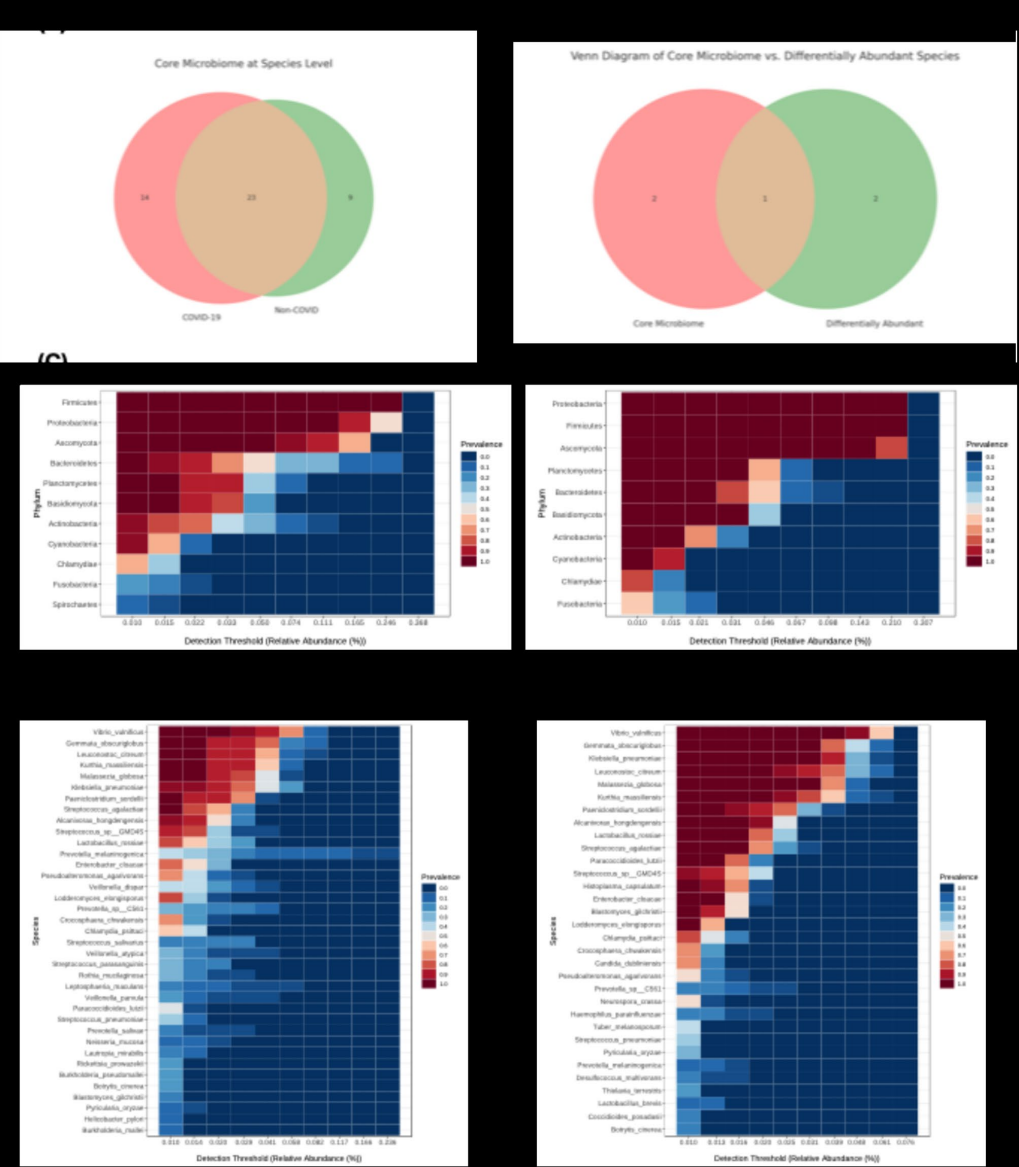
Core Microbiome Composition in COVID-19 vs Non-COVID Individuals: Figure presents the core microbiome composition at the species and phylum levels in COVID-19 and non-COVID individuals. (**A**) Venn diagram showing the overlap of core microbiome species between COVID-19 and non-COVID groups. (**B**) Venn diagram comparing core microbiome species with differentially abundant species. (**C**) Core microbiome at the phylum level for COVID-19 patients. (**D**) Core microbiome at the phylum level for non-COVID individuals. (**E**) Core microbiome at the species level for COVID-19 patients. (**F**) Core microbiome at the species level for non-COVID individuals.

The Venn diagram (Fig. 6A) illustrates the overlap of core microbiome species between COVID-19 and non-COVID groups. The diagram shows that 23 species are common to both groups, while 14 species are unique to the COVID-19 group and 9 species are unique to the non-COVID group. This overlap indicates that while there is a shared core microbiome, COVID-19 patients also possess unique microbial species not found in non-COVID individuals. The Venn diagram (Fig. 6B) compares the core microbiome species with differentially abundant species identified in previous analyses. The diagram reveals that some species within the core microbiome are also differentially abundant, suggesting that these key species play a significant role in differentiating the microbiomes of COVID-19 and non-COVID individuals. Specifically, one species is shared between the core microbiome and differentially abundant species, indicating its importance in the context of COVID-19^56^.

The Phylum Core Microbiome for COVID-19 patients (Fig. 6C) shows the distribution of key phyla that constitute the core microbiome in this group. The dominant phyla include *Firmicutes, Proteobacteria*, and *Bacteroidetes*. The presence of these phyla indicates their essential role in the oral microbiome of COVID-19 patients. The Phylum Core Microbiome for non-COVID individuals (Fig. 6D) highlights the distribution of core phyla in this group. Similar to COVID-19 patients, *Firmicutes* and *Bacteroidetes* are dominant, but there is a higher relative abundance of *Bacteroidetes* compared to COVID-19 individuals. This difference in phylum distribution suggests potential microbial shifts due to COVID-19. The Species Core Microbiome for COVID-19 patients (Fig. 6E) identifies key species that constitute the core microbiome in this group. Species such as *Streptococcus mutans* and *Veillonella parvula* are prominent, indicating their significant presence and potential role in the oral microbiome of COVID-19 patients. The Species Core Microbiome for non-COVID individuals (Fig. 6F) shows the key species in this group. The core species include *Prevotella melaninogenica* and *Rothia mucilaginosa*, which are more abundant in non-COVID individuals compared to COVID-19 patients. These differences at the species level highlight the unique microbial signatures associated with COVID-19^57^.

### Differential abundance of oral microbiome taxa

**Fig. 7:**
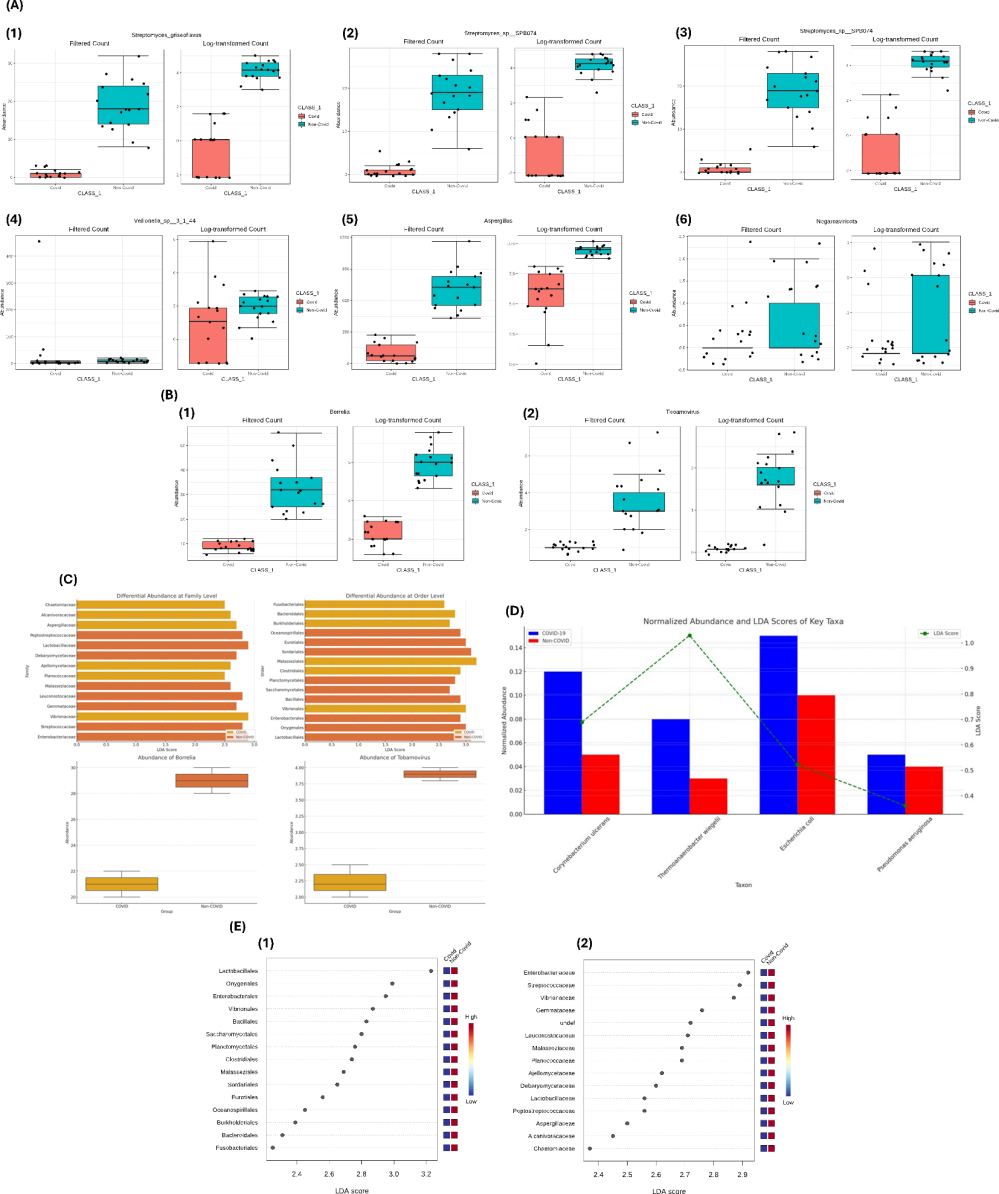
Differential Abundance of Microbial Taxa in COVID-19 and Non-COVID Groups: Figure presents the differential abundance analysis of microbial taxa between COVID-19 and non-COVID groups. (**A**) Box plots for the differential abundance of specific taxa between COVID-19 and non-COVID groups. A.1 shows the abundance of *Streptomyces griseoflavus*, A.2 and A.3 display the abundance of *Streptomyces sp. SPB074* in two different representations, A.4 highlights *Veillonella sp. 3_1_44*, Fig. A.5 illustrates the abundance of *Aspergillus*, and Fig. 7A.6 depicts the abundance of *Negarnaviricota*. (**B**) Box plots for the abundance of specific taxa, with B.1 showing the abundance of *Borrelia* and B.2 illustrating the abundance of *Tobamovirus* across the two groups. (**C**) Differential abundance at the family and order levels through an Linear Discriminant Analysis Effect Size (LEfSe) plot, highlighting the taxa that are significantly different between COVID-19 and non-COVID groups. (**D**) Combined normalized abundance and LDA (Linear Discriminant Analysis) scores of key taxa, emphasizing the differences in microbial abundance and their statistical significance between the groups. (**E**) LDA scores at different taxonomic levels: E.1 shows the LDA scores by order and E.2 shows the LDA scores by family, illustrating the key microbial taxa that distinguish the COVID-19 group from the non-COVID group.

In the differential abundance analysis, multiple steps were applied to ensure statistical robustness. The Benjamini–Hochberg correction was used to adjust p-values across multiple taxa comparisons, reducing the likelihood of false positives.^32^. The Benjamini–Hochberg correction method was chosen to reduce the likelihood of false positives across multiple taxa comparisons^33^. Additionally, multivariable regression models accounted for confounding factors such as age, gender, and tobacco and alcohol use, allowing for a more accurate interpretation of microbial differences between COVID-19 and non-COVID groups. The box plots in Fig. 7A reveal significant variations in the abundance of specific microbial taxa between COVID-19 and non-COVID groups. The abundance of *Streptomyces griseoflavus* (Fig. 7A.1) is notably higher in the non-COVID group, suggesting a potential suppression or reduction in this taxon due to COVID-19 infection. Similarly, *Streptomyces sp. SPB074* (Fig. 7A.2 and A.3) shows a marked decrease in abundance in the COVID-19 group compared to the non-COVID group. This trend may indicate that certain *Streptomyces* species are adversely affected by the presence of the virus. *Veillonella sp. 3_1_44* (Fig. 7A.4) exhibits a significant increase in abundance in the COVID-19 group, pointing towards a possible opportunistic expansion or a shift in microbial community dynamics favoring this species in COVID-19 patients. The abundance of *Aspergillus* (Fig. 7A.5) is also higher in the COVID-19 group, which could be related to secondary fungal infections or immune system dysregulation associated with COVID-19. Lastly, *Negarnaviricota* (Fig. 7A.6) shows a higher abundance in the COVID-19 group, suggesting a potential link between viral infections and changes in the abundance of certain viral taxa.

Figure 7B.1 and B.2 present the abundance of *Borrelia* and *Tobamovirus* respectively. The COVID-19 group shows a higher abundance of both taxa, with *Borrelia* (Fig. 7B.1) demonstrating a more pronounced increase. This may indicate a disruption in microbial homeostasis, potentially allowing for the proliferation of these taxa. *Tobamovirus* (Fig. 7B.2) also shows higher abundance in the COVID-19 group, highlighting the complex interactions between viral infections and the microbiome. The LEfSe plot (Fig. 7C) identifies taxa that are significantly different between the COVID-19 and non-COVID groups at both the family and order levels. Key taxa that distinguish the COVID-19 group include members of the orders *Lactobacillales* and *Clostridiales*, which are significantly more abundant in the non-COVID group. This analysis underscores the broad impact of COVID-19 on the oral microbiome, affecting various taxa across different taxonomic levels^49^. Figure 7D integrates normalized abundance and LDA scores of key taxa, providing a comprehensive view of the microbial differences between groups. This combined analysis reveals that taxa such as *Corynebacterium ulcerans* and *Escherichia coli* are significantly more abundant in the COVID-19 group, with high LDA scores indicating their strong association with COVID-19. Conversely, *Thermoanaerobacter wiegelii* and *Pseudomonas aeruginosa* show lower abundance and negative LDA scores, suggesting their decreased prevalence in COVID-19 patients. Figures 7E.1 and E.2 depict the LDA scores by order and family, respectively. The LDA scores highlight the key microbial taxa that are differentially abundant between COVID-19 and non-COVID groups. Orders such as *Lactobacillales* and *Clostridiales* (Fig. 7E.1) and families such as *Lactobacillaceae* and *Enterobacteriaceae* (Fig. 7E.2) show significant differences, reinforcing the impact of COVID-19 on the oral microbiome at multiple taxonomic levels. To adjust for potential confounding factors such as age, gender, and tobacco use, we utilized multivariable regression models in the differential abundance analysis, thereby enhancing the accuracy of the observed microbial differences between COVID-19 and non-COVID groups^58^.

The comparative analysis of the oral microbiome in COVID-19 patients and healthy individuals reveals significant differences across multiple dimensions. COVID-19 patients exhibit distinct microbial profiles characterized by altered abundance of key microbial taxa, reduced alpha diversity, and significant shifts in beta diversity. The core microbiome analysis indicates both shared and unique microbial species between the groups, with COVID-19 patients showing unique taxa that may contribute to disease pathology. Differential abundance analysis identifies specific taxa that are significantly more or less abundant in COVID-19 patients, underscoring the impact of the disease on the oral microbiome. These findings enhance our understanding of the microbiome’s role in COVID-19 and suggest potential targets for therapeutic intervention and disease management.

## Discussion

This study hypothesizes that COVID-19 impacts the oral microbiome uniquely due to its role as a primary entry point and interaction site for SARS-CoV-2. Unlike the more widely studied respiratory and gut microbiomes, the oral microbiome may provide early insights into systemic impacts of COVID-19 through shifts in microbial diversity and composition. The differential abundance analysis reveals that specific microbial taxa are significantly altered in COVID-19 patients. Notably, *Streptomyces griseoflavus* and *Streptomyces sp. SPB074* show a marked decrease in COVID-19 patients, suggesting that these beneficial bacteria are adversely affected by the viral infection. Conversely, *Veillonella sp. 3_1_44* and *Aspergillus* exhibit increased abundance in COVID-19 patients, indicating a possible opportunistic expansion due to the disrupted oral environment^49^. The presence of *Negarnaviricota* in higher abundance in COVID-19 patients further underscores the complex interactions between viral infections and the oral microbiome^59^.

While our study identifies distinct microbial shifts in COVID-19 patients, these findings reflect association rather than causation. Further longitudinal and experimental studies are required to determine whether these microbial changes directly contribute to post-COVID-19 pathology or are secondary to the infection. Our study highlights significant shifts in the overall microbial diversity and composition in COVID-19 patients. The alpha diversity measures, including Shannon, Chao1, and Simpson indices, consistently show reduced microbial diversity and evenness in COVID-19 patients compared to healthy individuals^3^. This reduction in diversity is indicative of a less resilient and more dysbiotic microbial community, which could have implications for oral and systemic health^4^. The beta diversity analysis, supported by PERMANOVA and ANOSIM statistical tests, confirms the distinct separation between the microbial communities of COVID-19 patients and healthy individuals, underscoring the significant impact of COVID-19 on the oral microbiome^60^. The core microbiome analysis reveals that while there are shared microbial species between COVID-19 and non-COVID groups, there are also unique taxa associated with each group. COVID-19 patients exhibit unique microbial species that may play a role in disease pathology and could serve as potential biomarkers for COVID-19^61^. The differential abundance analysis further identifies specific taxa, such as *Corynebacterium ulcerans* and *Escherichia coli*, which are significantly more abundant in COVID-19 patients and may be indicative of the altered microbial landscape associated with the disease^62^.

Our findings are consistent with previous studies that have reported changes in the oral microbiome due to COVID-19. For example, a study found similar reductions in microbial diversity in COVID-19 patients, while another research observed increased abundance of opportunistic pathogens such as *Pseudomonas aeruginosa*^63,64^. These comparisons highlight the robustness of our results and underscore the broader applicability of our findings. The observed microbial changes could be driven by several mechanisms. SARS-CoV-2's interaction with ACE2 receptors in the oral cavity might directly influence the local microbiome^65^. Additionally, the systemic inflammatory response associated with COVID-19 could create a hostile environment for beneficial microbes, while providing niches for opportunistic pathogens to thrive^66^. Further research is needed to elucidate these mechanisms in detail.

Understanding the changes in the oral microbiome associated with COVID-19 can lead to the identification of microbial markers for early diagnosis and monitoring of the disease. This knowledge can also inform the development of microbiome-based therapeutic interventions aimed at restoring microbial balance and improving patient outcomes. For instance, targeted probiotics or prebiotics could be designed to enhance beneficial bacteria and suppress opportunistic pathogens, thereby mitigating the impact of COVID-19 on the oral and systemic health of patients^67^. Future studies should focus on longitudinal analysis to capture the dynamic changes in the oral microbiome throughout the course of COVID-19. Additionally, functional analysis using Metatranscriptomics or Metabolomics could provide deeper insights into the metabolic and functional alterations associated with COVID-19^68^. Collaborative efforts with clinical researchers could facilitate the translation of these findings into practical therapeutic interventions.

### Limitations of this study

Despite these significant findings, this study has several limitations. The sample size is relatively small, which may affect the generalizability of the results. The cross-sectional nature of the study limits our ability to capture dynamic changes in the oral microbiome over time^69^. Additionally, we did not perform functional analysis, such as Metatranscriptomics or Metabolomics, which could provide deeper insights into the metabolic and functional alterations associated with COVID-19^70^. These findings, though promising for public health insights, are preliminary. Before clinical applications, further studies are essential to validate microbial markers and explore their role in diagnostics and interventions, ensuring robust and reliable public health strategies for COVID-19 and similar viral diseases.

## Conclusion

Overall, this study presents a comprehensive comparative analysis of the oral microbiome in COVID-19 patients and healthy individuals. Our findings reveal significant alterations in microbial diversity, composition, and abundance associated with COVID-19. These changes highlight potential microbial diagnostic and therapeutic markers for managing the disease. While the study provides valuable insights into the impact of COVID-19 on the oral microbiome, further research is necessary to explore the functional implications of these microbial shifts and to develop effective microbiome-based interventions for improving patient outcomes. This research represents an important step towards integrating microbiome science into the clinical management of COVID-19.

## Electronic supplementary material

Below is the link to the electronic supplementary material.

Supplementary Material 1

## Data Availability

The datasets generated and/or analysed during the current study are available in the NCBI (SRA) repository, accession number PRJNA801862.

## Abbreviations

COVID-19: Coronavirus Disease 2019
SARS-CoV-2: Severe acute respiratory syndrome coronavirus 2
ACE2: Angiotensin-converting enzyme 2
RNA: Ribonucleic acid
cDNA: Complementary DNA
DNA: Deoxyribonucleic acid
ONT: Oxford Nanopore Technologies
PCR: Polymerase chain reaction
QIAamp: QIAGEN’s DNA extraction product line
HS: High sensitivity
GUI: Graphical user interface
GRCh38: Genome reference consortium human build 38
RLE: Relative log expression
CSS: Cumulative sum scaling
FitZIG: Zero-inflated Gaussian
RefSeq: Reference sequence
IQR: Inter-quantile range
TMM: Trimmed mean of M-values
PERMANOVA: Permutational multivariate analysis of variance
PCoA: Principal coordinate analysis
ANOSIM: Analysis of similarity
LDA: Linear discriminant analysis
LEfSe: Linear discriminant analysis effect size
