# Supplementary material for: Comparative metagenomic analysis of the oral microbiome in COVID-19 patients and healthy individuals
Source: Sci Rep. 2025 Mar 25;15:10303. doi: 10.1038/s41598-024-81864-3 (PMC11937335; doi:10.1038/s41598-024-81864-3)
Supplement: Supplementary file 1 — Supplementary Material 1 [file 41598_2024_81864_MOESM1_ESM.pdf]

Patient ID	AGE	GENDER	TOBACCO (YES/NO)	ALCOHOL (YES/NO)	Current Diseases (YES/NO)	DISEASE STATE	COMORBIDITIES	Medication (YES/NO)
C1	36	Male	YES	YES	Yes	Covid-Mild	Vasculitic Neuropathy,Budd chiari Syndrome,Sensory Ataxia	YES
C10	30	Male	NO	NO	Yes	Covid-Severe	NIL	NO
C11	30	Female	NO	NO	Yes	Covid- Mild	NIL	NI
C12	46	Female	NO	NO	Yes	Covid-Mild	CHD,Viral Meningitis	YES
C2	19	Female	NO	NO	Yes	Covid-Mild	NIL	NO
C3	32	Female	NO	NO	Yes	Covid-Mild	NIL	NO
C5	31	Female	NO	NO	Yes	Covid- Mild	NIL	NO
C4	31	Female	NO	NO	Yes	Covid-Mild	NIL	NO
C6	34	Female	NO	NO	Yes	Covid-Mild	NIL	NO
C7	34	Female	NO	NO	Yes	Covid- Mild	NIL	NO
C8	36	Male	NO	NO	Yes	Covid- Mild	Mutism,Since Birth,S/P LSCS-9 months Back	NO
C9	58	Female	NO	NO	Yes	Covid- Mild	NIL	NO
C13	49	Male	NO	NO	Yes	Covid-Mild/Diabetic	NIL	NO
C14	63	Male	NO	NO	Yes	Covid-Moderate/Diabetic	NIL	NO
C15	63	Male	NO	NO	Yes	Covid- Moderate/Diabetic	NIL	NO
C16	42	Female	NO	NO	Yes	Covid-Mild/Diabetic	S/P MVR WITH LA	YES
C17	69	Male	NO	NO	Yes	Covid- Moderate/Diabetic	HTN,CAD	YES
C18	88	Female	NO	NO	Yes	Covid- Moderate/Diabetic	HTN	YES
C19	67	Female	NO	NO	Yes	Covid- Moderate/Diabetic	HTN	YES
C20	53	Male	NO	NO	Yes	Covid-Mild/Diabetic	NIL	YES
C21	48	Male	NO	NO	Yes	Covid-Mild/Diabetic	NIL	YES
C22	42	Male	NO	NO	Yes	Covid- Mild/Diabetic	NIL	NO
C23	36	Male	NO	NO	Yes	Covid-Mild/Diabetic	NIL	NO
C24	66	Male	NO	NO	Yes	Covid-Mild/Diabetic	NIL	NO
NC1	18	Male	NO	NO	No	Healthy	NIL	NO
NC2	39	Male	NO	NO	No	Healthy	NIL	NO
NC3	39	Male	NO	NO	No	Healthy	NIL	NO
NC4	29	Male	NO	NO	No	Healthy	NIL	NO
NC5	53	Female	NO	NO	No	Healthy	NIL	NO
NC6	26	Male	YES	YES	No	Healthy	NIL	NO
NC7	23	Female	NO	NO	No	Healthy	NIL	NO
NC8	35	Female	NO	NO	No	Healthy	NIL	NO
NC9	25	Female	NO	NO	No	Healthy	NIL	NO
NC10	24	Male	NO	NO	No	Healthy	NIL	NO
NC11	24	Male	NO	NO	No	Healthy	NIL	NO
NC12	34	Male	NO	NO	No	Healthy	NIL	NO
NC13	37	Male	NO	NO	Yes	Diabetic	HTN	YES
NC14	56	Male	NO	NO	Yes	Diabetic	CAD, BP, Thyroiddisease	YES
NC15	48	Male	NO	NO	Yes	Diabetic	HTN, CAD	YES
NC16	51	Female	NO	NO	Yes	Diabetic	Hypothyrodism, BP, Heart disease(CAD),Obese class1,	YES
NC17	46	Male	NO	NO	Yes	Diabetic	CAD	YES
NC18	39	Female	NO	NO	Yes	Diabetic	NIL	NO
NC19	68	Male	NO	NO	Yes	Diabetic	CAD	YES
NC20	46	Male	NO	NO	Yes	Diabetic	CABG done recently	YES
NC21	54	Female	NO	NO	Yes	Diabetic	HTN	YES
NC22	47	Male	NO	NO	Yes	Diabetic	Thyroid disease 4y	YES
NC23	57	Male	NO	NO	Yes	Diabetic	HTN	YES
NC24	69	Female	NO	NO	Yes	Diabetic	HTN, Thyroid disease, CAD	YES
